# Immunomodulating Effect of the Consumption of Watercress *(Nasturtium officinale)* on Exercise-Induced Inflammation in Humans

**DOI:** 10.3390/foods10081774

**Published:** 2021-07-30

**Authors:** Hendrik Schulze, Johann Hornbacher, Paulina Wasserfurth, Thomas Reichel, Thorben Günther, Ulrich Krings, Karsten Krüger, Andreas Hahn, Jutta Papenbrock, Jan P. Schuchardt

**Affiliations:** 1 Institute of Food Science and Human Nutrition, Leibniz University Hannover, D-30167 Hannover, Germany; schulze@nutrition.uni-hannover.de (H.S.); wasserfurth@nutrition.uni-hannover.de (P.W.); hahn@nutrition.uni-hannover.de (A.H.); 2 Institute of Botany, Leibniz University Hannover, D-30419 Hannover, Germany; j.hornbacher@botanik.uni-hannover.de (J.H.); jutta.papenbrock@botanik.uni-hannover.de (J.P.); 3 Department of Exercise Physiology and Sports Therapy, Institute of Sport Science, Justus Liebig University Giessen, D-35394 Giessen, Germany; thomas.reichel@sport.uni-giessen.de (T.R.); karsten.krueger@sport.uni-giessen.de (K.K.); 4 Institute of Food Chemistry, Leibniz University Hannover, D-30167 Hannover, Germany; thorben.detering@lci.uni-hannover.de (T.G.); krings@lci.uni-hannover.de (U.K.)

**Keywords:** watercress, cruciferous vegetables, glucosinolates, gluconasturtiin, anti-inflammatory, pro-inflammatory

## Abstract

The vegetable watercress (*Nasturtium officinale* R.Br.) is, besides being a generally nutritious food, a rich source of glucosinolates. Gluconasturtiin, the predominant glucosinolate in watercress, has been shown to have several health beneficial properties through its bioactive breakdown product phenethyl isothiocyanate. Little is known about the immunoregulatory effects of watercress. Moreover, anti-inflammatory effects have mostly been shown in in vitro or in animal models. Hence, we conducted a proof-of-concept study to investigate the effects of watercress on the human immune system. In a cross-over intervention study, 19 healthy subjects (26.5 ± 4.3 years; 14 males, 5 females) were given a single dose (85 g) of fresh self-grown watercress or a control meal. Two hours later, a 30 min high-intensity workout was conducted to promote exercise-induced inflammation. Blood samples were drawn before, 5 min after, and 3 h after the exercise unit. Inflammatory blood markers (IL-1β, IL-6, IL-10, TNF-α, MCP-1, MMP-9) were analyzed in whole blood cultures after ex vivo immune cell stimulation via lipopolysaccharides. A mild pro-inflammatory reaction was observed after watercress consumption indicated by an increase in IL-1β, IL-6, and TNF-α, whereas the immune response was more pronounced for both pro-inflammatory and anti-inflammatory markers (IL-1β, IL-6, IL-10, TNF-α) after the exercise unit compared to the control meal. During the recovery phase, watercress consumption led to a stronger anti-inflammatory downregulation of the pro-inflammatory cytokines IL-6 and TNF-α. In conclusion, we propose that watercress causes a stronger pro-inflammatory response and anti-inflammatory counter-regulation during and after exercise. The clinical relevance of these changes should be verified in future studies.

## 1. Introduction

With the revival of domestic greens watercress *(Nasturtium officinale* R.Br.), a member of the Brassicaceae family, gains a growing interest in science. The semi-aquatic plant species native to Europe and Asia is often consumed as a salad or garnish, or as part of a soup, especially in the Mediterranean kitchen. It is valued for its high nutrient density caused by a low energy content and high amounts of vitamins (B1, B2, B3, B6, C, E), minerals (calcium, iron), and phytochemicals (polyphenols, terpenes) [1,2,3]. Like all members of the Brassicaceae family, watercress contains mustard oil glycosides or glucosinolates (GLS), of which gluconasturtiin is the predominant GLS in watercress. As a precursor, it is converted into the bioactive compound phenethyl isothiocyanate (PEITC) upon tissue disruption due to the action of the thioglucosidase myrosinase.

Several studies investigated the health beneficial effects of watercress and PEITC including antioxidative, anti-inflammatory, antidiabetic, anti-allergic, antibacterial, hypolipemic, cardioprotective, and anticancer effects (reviewed in [2]). While most of these effects have been observed in vitro or in animal studies, only a few human intervention studies with watercress have been carried out. Moreover, human intervention studies that administered watercress have mainly focused on antioxidative [4,5] and anticancer effects [6]. The influence of watercress on the immune system, in particular anti-inflammatory activity, has barely been investigated in human studies thus far. There has yet been no confirmation that watercress and its ingredients gluconasturtiin/PEITC act in a similar way in humans compared to effects observed in vitro, namely, by inhibiting the pro-inflammatory nuclear factor kappa B (NfκB) pathway [7]. Because the NfκB pathway can be stimulated directly by reactive oxygen species (ROS) [8] or indirectly by the ROS-dependent heat shock response [9,10], antioxidants might attenuate the exercise-induced inflammation [11]. As a consequence, it is necessary to determine the levels of antioxidants and their capacity in watercress. Moreover, it remains unknown as to whether other gluconasturtiin metabolites are formed in vivo and how they contribute to an antioxidative effect such as that indicated for benzenepropanenitrile [12].

We performed a pilot study with four subjects to examine the effect of a single dose of fresh watercress on various biomarkers of exercise-induced inflammation [13]. On the basis of the results of that previous study, where we observed indications for anti-inflammatory effects, we conducted this follow-up study with a greater number of subjects to further characterize the inflammatory response of watercress consumption. After consuming 85 g of fresh watercress, untrained subjects had to complete a high-intensity workout to induce a pro-inflammatory condition. Inflammatory blood markers (IL-1β, IL-6, IL-10, TNF-α, MCP-1, MMP-9) were analyzed in whole blood cultures after ex vivo immune cell stimulation via lipopolysaccharides (LPS).

## 2. Materials and Methods

### 2.1. Plant Material

The administered watercress *(Nasturtium officinale)* was obtained from the Institute of Botany, Leibniz University Hannover. Cuttings were taken for propagation and cultivated in a hydroponic greenhouse system using a Hoagland solution. After 8 weeks, the plant material was harvested freshly on a daily basis. Following a 45 min wet transport, it was cut as little as necessary for consumption.

### 2.2. Analysis of Glucosinolates by HPLC/LC–MS

GLS were analyzed by HPLC–UV according to Hornbacher et al. [14]. The GLS content of the watercress samples were measured in triplicate. All standard substances were checked for identity. For the identification of the GSL in *N. officinale*, samples were analyzed by liquid chromatography–mass spectrometry (LC–MS). A volume of 10 μL was injected into the HPLC system (Shimadzu, Darmstadt, Germany) and separated on a Knauer Vertex Plus column (250 × 4 mm, 5 μm particle size, packing material ProntoSIL 120-5 C18-H) equipped with a pre-column (Knauer, Berlin, Germany). A water (solvent A)–methanol (solvent B), both containing 2 mM ammonium acetate, gradient was used with a flow rate of 0.8 mL/min at 30 °C. For measuring the samples, the following gradient was used: 10–90% B for 35 min, 90% for 2 min, 90–10% B for 1 min, and 10% B for 2 min. Detection of the spectra in the range 190–800 nm was performed with a diode array detector (SPD-M20A, Shimadzu, Darmstadt, Germany). The HPLC system was coupled to an AB Sciex TripleTOF mass spectrometer (AB Sciex TripleTOF 4600, Canby, OR, USA). At a temperature of 600 °C and an ion spray voltage floating of −4500 V, the negative electrospray ionization (ESI) was performed. For the ion source gas one and two 50 psi were used and for the curtain gas 35 psi. In the range of 100–1500 Da in the TOF range, the mass spectra as well as the MS/MS spectra from 150–1500 Da at a collision energy of −10 eV were recorded. Peaks were identified by analyzing the characteristic mass fragments of ds-4-methoxyglucobrassicin (195, 398, 433, 795) and ds-glucoarabishirsutain (195, 382, 417, 763). Due to lack of standards of the GSLs fractions of the measured samples were collected in a fraction collector (FRC-10A Shimadzu, Darmstadt, Germany), dried in a vacuum centrifuge, and dissolved in 300 μL ultrapure water. The retention time for every GSL was determined by measuring either the collected fraction or the authentic standard (Phytolab, Vestenbergsgreuth, Germany) with the HPLC system, as described above.

### 2.3. Measurement of Antioxidant Contents and Antioxidant Capacity

The measurements of carotenoid, total phenol and total flavonoid contents, as well as the measurement of the oxygen radical absorbance capacity (ORAC), were performed according to Boestfleisch et al. [15].

Tocopherol contents were analyzed according to Cruz et al. [16] with modifications. Small portions of finely ground fresh sample (1.0 g) were weighed accurately into amber glass vials containing ascorbic acid (50 mg), butylated hydroxytoluene (1 mg), and internal standard (1 µg *δ*-tocopherol). Samples were homogenized with methanol (2 mL) by vortex mixing for 1 min. Then, dichloromethane (4 mL) was added and vortex-mixed for 1 min. Subsequently, 0.9% (*w*/*v*) NaCl (1 mL) was added, the mixture was homogenized (1 min) and centrifuged (3 min, 14,000× *g*), and the clear lower layer was transferred to an amber flask. Extraction was repeated twice with dichloromethane. The extracts were combined and vacuum-dried in a vacuum centrifuge (Eppendorf, Hamburg, Germany) at 25 °C. The extract was recovered with 1 mL of *n*-hexane and anhydrous sodium sulfate was added (around 100 mg). After an additional centrifugation (5 min, 14,000× *g*), the supernatant was analyzed immediately. Analysis was performed with an HPLC system equipped with a Nucleodur C18 column (250 mm × 4.6 mm; Macherey-Nagel, Düren, Germany). Tocopherols were separated with an isocratic gradient consisting of 90% *n*-hexane and 10% diethyl ether at room temperature and a flow rate of 1 mL/min. Analytes were monitored with a fluorescence detector (Shimadzu, Duisburg, Germany). Excitation was performed at 289 nm and fluorescence of analytes was analyzed at 331 nm.

### 2.4. Human Study Design and Subjects

**Figure 1:**
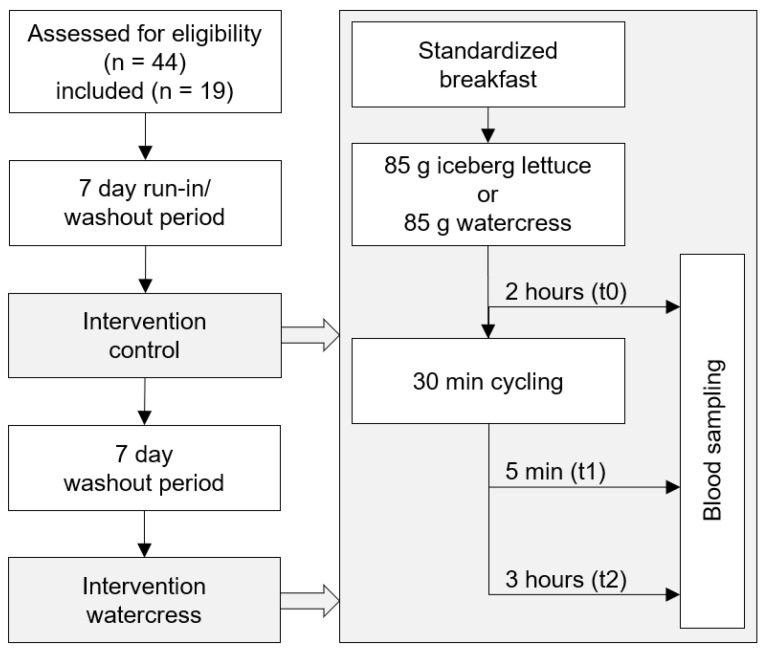
Flow diagram showing the timeline of the study.

An overview of the timeline of the study and the interventions in particular is shown in Figure 1. The inclusion criteria of the cross-over study were age between 18 and 35 years, BMI between 18 and 30 kg/m^2^, and less than two hours of moderate exercise per week, classifying these participants as untrained. For the questionnaire-based assessment of the training status, we factored in leisure time physical activities such as jogging or weight-lifting, as well as daily non-athletic exertions such as movement by foot or bike. The exclusion criteria were cardiovascular or metabolic disease, smoking, pregnancy, drug or alcohol dependency, concurrent participation in another clinical trial or in another study within the last 30 days, and intake of antioxidative or antiphlogistic medicine or dietary supplements.

As part of a run-in/washout phase, subjects refrained from consuming foods rich in polyphenols, vitamin C and E (mainly berries, nuts, and vegetables of the Brassicaceae family) seven days before each examination. To ensure compliance, participants received written instructions and a list of foods that should not be consumed. On the basis of the consumed amount in similar studies [4,5,13], subjects ate a single dose of 85 g of fresh watercress accompanied by a standard breakfast (two buns, cream cheese or oat spread, yoghurt or balsamic dressing). In the control group, 85 g of iceberg lettuce was administered instead. Two hours after consumption, the first blood sample (t0) was taken. Immediately after blood draw, the subjects completed a 30 min high-intensity endurance workout on echo bikes within a parameter range of 80–92% HR_max_, 120–145 W, and a final rating of perceived exertion of 17.8. Throughout the workout, heart rates were recorded using a heart rate monitor watch with a Bluetooth heart rate sensor chest strap (RC 14.11, Sigma-Elektro, Neustadt, Germany). Additional blood samples were taken 5 min (t1) and 3 h (t2) after the end of the exercise. The subjects were served a lunch in the meantime consisting of a potato soup. All blood samples were obtained by venipuncture of an arm vein using Multifly needles (Sarstedt, Nürnbrecht, Germany) into heparin plasma monovettes (Sarstedt, Nürnbrecht, Germany). To assure that subjects did not enter the examination with elevated inflammatory markers due to an infection, we determined C-reactive protein in serum using serum monovettes (Sarstedt, Nürnbrecht, Germany). Both interventions (control and watercress) were conducted identically with an intermediary washout phase of 7 days.

The study was carried out following the rules of the Declaration of Helsinki and was approved by the Ethics Committee at the Medical Chamber of Lower Saxony (30/37/2020, Hannover, Germany, 10/2020).

### 2.5. Measurement of Inflammatory Markers by Bio-Plex Multiplex Immunoassay

The freshly drawn blood samples were stimulated ex vivo in whole blood cultures via lipopolysaccharides (LPS). Therefore, samples were immediately diluted 1:5 with the cell culture medium RPMI 1640 including 20 mmol HEPES and L-glutamine (Sigma-Aldrich, Hamburg, Germany) and added antibiotics (100 U/mL penicillin and 100 µg/mL streptomycin; Sigma-Aldrich, Hamburg, Germany). The samples were seeded into 12-well microtiter plates and mixed with 10 ng/mL (final concentration) LPS from *Escherichia coli* (Sigma-Aldrich, Hamburg, Germany). The plates were incubated for 24 h at 37 °C without a CO_2_ application. The used HEPES buffer is able to stabilize the pH over 24 h. The supernatants were frozen at −80 °C until analysis.

The levels of the inflammatory markers (IL-1β, IL-6, IL-10, TNF-α, MCP-1, MMP-9) in whole blood culture supernatant were simultaneously determined using a human Magnetic Luminex Assay (Bio-Techne, Abingdon, Oxon, UK) and a Magpix Luminex instrument (Luminex Corp, Austin, TX, USA).

### 2.6. Data Analysis and Statistical Methods

Data are presented as the means ± standard deviation. All variables were tested for normal distribution by Shapiro–Wilk test. In the case of not normally distributed data, a suitable transformation was applied, and parametric tests were used. Differences among inflammatory markers were analyzed using ANOVA with repeated measures. In addition, groups were compared using a *t*-test for dependent means. To calculate correlations, we utilized Pearson correlation (parametric data) and Spearman’s rho correlation (non-parametric data). Statistical significance was regarded as values of *p* ≤ 0.05. Analyses were conducted using Infostat (version 2012; University of Córdoba, Córdoba, Argentine) and SPSS (version 27; SPSS Inc., Chicago, IL, USA).

## 3. Results

Of the 21 recruited subjects, 19 completed the study (Table 1). Incomplete study data were excluded from statistical analyses. One subject failed to participate because of illness. Another subject showed increased serum levels of C-reactive protein (>0.5 mg/L), indicating an elevated systemic inflammation. No health- or workout-related incidents occurred during the study, with the exception of one subject taking a two-minute break from the exercise due to total exhaustion.

### 3.1. Plant Material

#### 3.1.1. Levels of Glucosinolates

**Figure 2:**
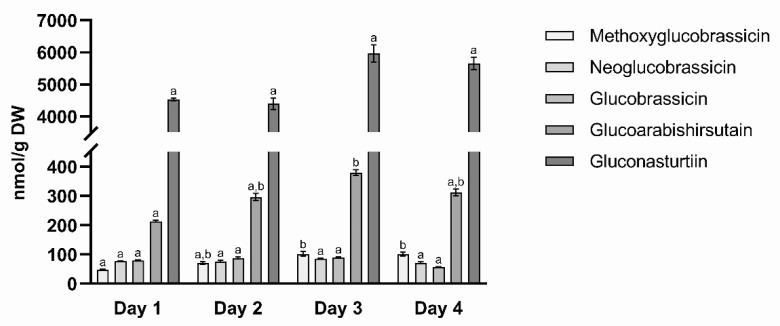
Mean concentration of different glucosinolates (GLS) (nmol/g DW) in watercress at different sampling times. The standard deviation represents the values for three technical replicates. Analysis of variance (ANOVA) was performed with Infostat. Means with a common letter are not significantly different.

Gluconasturtiin was recognized as the predominant GLS in watercress with a fraction of 90.5 ± 1.1% of the total GLS content. In addition, minor amounts of glucoarabishirsutain (5.3 ± 0.8%), glucobrassicin (1.4 ± 0.4%), neoglucobrassicin (1.4 ± 0.3%), and 4-methoxyglucobrassicin (1.4 ± 0.4%) were found (Figure 2, Table A1). In the course of the study, GLS contents varied notably with the most impactful difference in gluconasturtiin of 36% at day 3 compared to the previous day. This resulted in a difference of 34% of the total GLS content while maintaining the partial composition of the GLS profile.

#### 3.1.2. Levels of Antioxidants and Antioxidant Capacity

Contents of flavonoids as well as ascorbic acid in analyzed watercress were similar at all sampling days (Table A2). Contents of total phenols were slightly higher in samples taken at day 3 and day 4, whereas carotenoid contents were slightly higher at day 2 and day 4. Tocopherol contents as well as ORAC were similar at all sampling days.

### 3.2. Levels of Inflammatory Blood Markers

Statistical analysis using the ANOVA with repeated measures showed significant differences between the sampling times for all analyzed parameters (*p* ≤ 0.001). Hence, the acute exercise affected concentrations of all measured inflammatory markers. In addition, the same analysis pointed out that the consumption of watercress led to significant differences for the levels of IL-1β, IL-6, and IL-10 across all sampling times (IL-1β (*p* = 0.006), IL-6 (*p* = 0.006), IL-10 (*p* ≤ 0.001)). The remaining parameters showed no significant differences (TNF-α (*p* = 0.382), MCP-1 (*p* = 0.192), MMP-9 (*p* = 0.118)). With regards of the varying GLS levels of the plant material, no correlations with the inflammatory markers were found.

**Figure 3:**
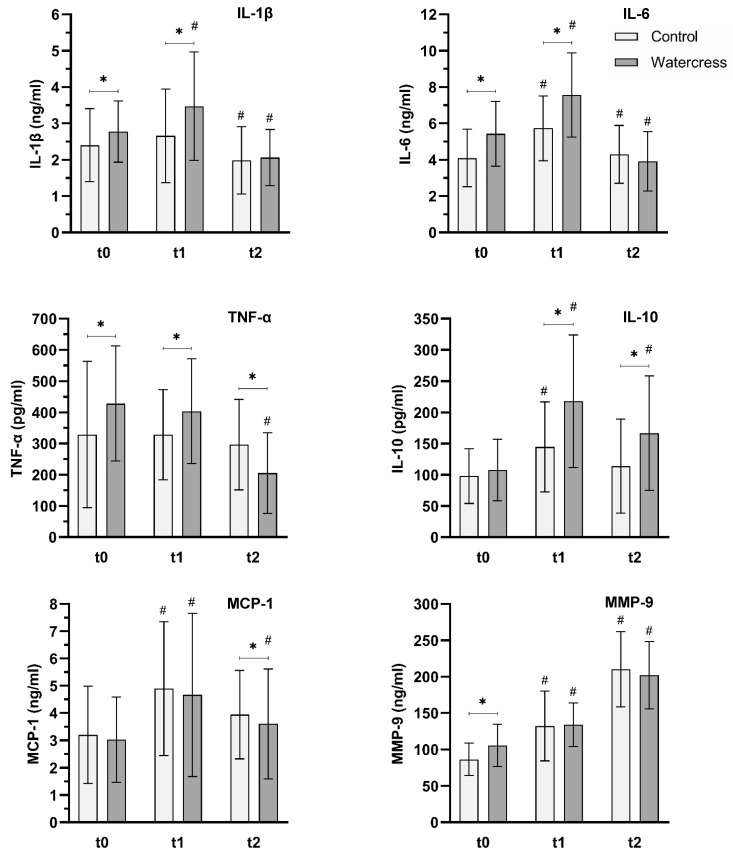
Effect of acute watercress consumption on blood markers of inflammation (IL-1β, IL-6, IL-10, TNF-α, MCP-1, MMP-9) in ex vivo LPS-stimulated whole blood cultures after high-intensity workout in untrained subjects. t0, pre-exercise; t1, 5 min post-exercise; t2, 3 h post-exercise. Analysis of variance (ANOVA) was performed with SPSS. * Significant difference between control and watercress. ^#^ Significant difference to previous sampling point.

**Figure 4:**
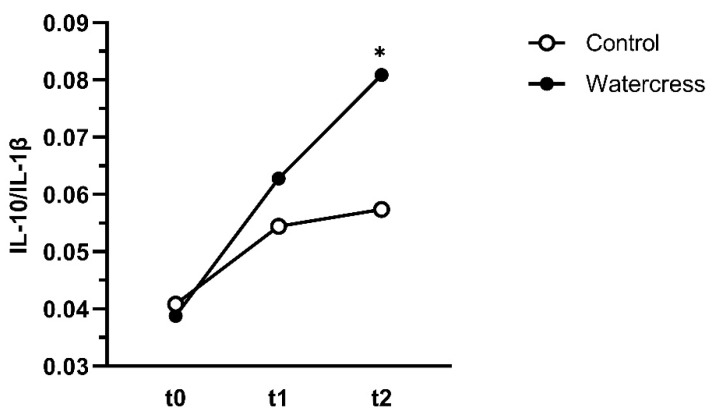
Effect of acute watercress consumption on the ratio of IL-10 (anti-inflammatory) and IL-1β (pro-inflammatory). t0, pre-exercise; t1, 5 min post-exercise; t1, 3 h post-exercise. Analysis of variance (ANOVA) was performed with SPSS. * Significant difference between control and watercress.

Significantly higher concentrations of the pro-inflammatory cytokines IL-1β (16%), IL-6 (33%), and TNF-α (30%), as well as the enzyme MMP-9 (22%), were observed in the watercress group compared to the control group two hours after the watercress consumption (t0) (Figure 3, Table A3). Upon exercise-stimulation (t1), the control group showed a significant rise in all inflammatory markers, specifically IL-6 (33%), IL-10 (47%), MCP-1 (53%), and MMP-9 (53%) with IL-1β and TNF-α showing no reaction. Compared to the control breakfast, levels of IL-1β, IL-6, IL-10, and TNF-α were significantly higher after the watercress breakfast (31%, 32%, 51%, and 23%, respectively). To determine if the consumption of watercress resulted in a stronger increase of the cytokines regardless of the pre-exercise levels, we compared the differences (t1 − t0). Thereby, a significant stronger upregulation of the anti-inflammatory cytokine IL-10 was found. After the recovery phase (t2), all inflammatory markers except MMP-9 decreased. A comparison of the differences (t2 − t1) between the watercress and the control group revealed a significantly stronger downregulation of the pro-inflammatory cytokines IL-6 and TNF-α and a trend in IL-1β (*p* = 0.062). In the case of TNF-α, the level at t2 was even lower compared to the pre-exercise state of the control group. This post-exercise downregulation can also be observed in the ratio of the anti-inflammatory IL-10 and the pro-inflammatory IL-1β (Figure 4). The watercress consumption influenced the ratio 3 h after exercise towards the anti-inflammatory reaction.

## 4. Discussion

Many food compounds are known to assure the maintenance of the immune system or improve its performance [17]. By interacting with ROS or affecting cytokine biology, their intake can modulate immune function. A wide variety of anti-inflammatory nutrients has been investigated thus far. While many act as antioxidants and, hence, indirectly modulate the ROS-dependent NfκB activation [18] such as ascorbic acid, glutathione, or carotenoids, others operate as pro-resolving mediators such as omega-3 fatty acids [19].

A common misconception is that pro-inflammatory processes at all times need to be annihilated to prevent the body from harm. Similar to oxidative stress, pro-inflammatory processes can have both detrimental and beneficial effects to the human body. When inflammatory processes become chronic, they often have negative impacts. Chronic low-grade inflammation is associated with a wide range of chronic conditions, such as the metabolic syndrome, cardiovascular disease, type 2 diabetes, and non-alcoholic fatty liver disease. However, if the body is able to resolve inflammatory processes, they can provide signals for adaptation in physiological contexts such as sport [20]. In its acute form, it is generally a beneficial procedure, which removes stimuli and initiates the repair system. As the first line of defense, pro-inflammatory cytokines such as IL-1β, IL-6, and TNF-α promote the activation and secretion of more cytokines and acute-phase proteins as well as the proliferation and differentiation of T- and B-cells. The time-delayed increase of the cytokine IL-10 has been widely recognized as a suppression of the inflammatory response by its downregulating effects on TNF-α and IL-1 [21,22,23]. The immune system acts through a fluctuation between a pro-inflammatory response and an anti-inflammatory or inflammation resolving counter-regulation. An increase in the magnitude of the fluctuation could be interpreted as positive for the capacity of the system. Thus, a mild activation of the immune system through dietary components might aid in resolving inflammation by a preceding mobilization. We assume that this immunomodulating effect also applies to watercress and its ingredients.

Upon exercise- and LPS-stimulation, a solid, non-excessive immune reaction was observed in IL-6 and IL-10, with a greater response after the watercress consumption. Interestingly, the subsequent inflammatory counter-regulation of IL-6, TNF-α, and IL-1β was more pronounced in the watercress group, which likely was a result of the higher exercise-induced levels of IL-10. This shift towards an anti-inflammatory response after the watercress consumption was supported by the increased ratio of IL-10 and IL-1β. Our results suggest that watercress intake stimulates the immune system, which might enhance its metabolic capacity. The finding that the consumption of watercress causes an initial pro-inflammatory reaction followed by a greater exercise-induced response in both pro- and anti-inflammatory markers is remarkable and has not been observed thus far. We assume that gluconasturtiin, in particular PEITC, is responsible for the observed immunomodulating effect, where PEITC might operate as an activator in T-lymphocytes or macrophages or their receptors and thereby stimulates or sensitizes the cytokine production. It is also conceivable that PEITC activates the heat shock response, which leads to a stimulation of the pro-inflammatory NfκB pathway [24]. Another explanation is that specific components of watercress might interfere with the immunometabolism and thereby stimulate its function. Although the average contents of secondary metabolites besides gluconasturtiin and the ORAC are in the lower range of vegetables, it is likely that the observed effects are mainly caused by the GLS. The extraction procedure for the evaluation of the ORAC uses methanol as organic solvent, which inhibits the hydrolysis of gluconasturtiin to PEITC. However, PEITC would contribute only very little to the overall ORAC, since its capacity to scavenge ROS was reported to be 1.9 µg TE/mg PEITC [25]. The levels of gluconasturtiin in the obtained watercress resemble the results of the pilot study [13] and were in the same range of reported contents for raw watercress [26].

In contrast to our results, PEITC and watercress extracts have thus far been shown to possess only anti-inflammatory and no pro-inflammatory properties [27,28,29,30,31]. The pilot study, on which this investigation is based on, aligns with these observations [13]. In vitro studies presume the mechanism behind this effect of PEITC in the inhibition of the pro-inflammatory NfκB pathway in macrophages, possibly through the modulation of toll-like receptors [31,32,33]. Due to the redox-sensitivity of NfκB, the activation of the antioxidative nuclear factor erythroid 2-related factor 2 (Nrf2) pathway by PEITC plays a considerable role in its anti-inflammatory effect [34]. Previous human intervention studies have focused mainly on those antioxidative effects of watercress. They showed that a regular consumption as well as a single dose of watercress reduces the oxidative stress in various biomarkers [4,5]. On the basis of the antioxidative and thereby assumed anti-inflammatory effects, the outcome of this study does not align with the previous literature. Although PEITC is able to promote oxidative stress at very high concentrations, presumably acting as a scavenger for glutathione [35], it remains debatable as to whether a single dose of 85 g of watercress provides the body with the necessary range of concentration. In consequence of the redox-sensitivity of NfκB, an initial pro-oxidative effect after the consumption of watercress could explain the simultaneously increased cytokine levels of IL-1β, IL-6, and TNF-α. The pro-oxidative state would be further enhanced by the exercise, which subsequently results in an even more pronounced pro-inflammatory response in the watercress group and a stronger induction of the counter-regulatory Nrf2-mediated antioxidant response that is observed in the recovery phase.

## 5. Conclusions

The course of inflammatory markers after the watercress consumption with initially increasing pro-inflammatory markers and a higher release of an anti-inflammatory marker in the recovery phase has not yet been described in the literature. We interpret this observation with a mild activation of the immune system resulting in a stronger pro-inflammatory reaction, which is more effectively resolved by a powerful anti-inflammatory counter-regulation. This thesis and the clinical relevance have to be investigated in future studies. Moreover, cell culture studies are necessary to explore the underlying effects of watercress components on leukocytes. Likewise, it must not be ignored that the immune cells were stimulated ex vivo by LPS. The results and the possible effects on the immune system can therefore not be directly transferred to the situation in vivo. Because watercress is a complex food with considerable amounts of other potentially immunomodulating substances besides PEITC, a causal relationship between the observed effects and gluconasturtiin/PEITC cannot be stated. A clinical trial with isolated gluconasturtiin/PEITC should be conducted in order to confirm the effect and whether its magnitude is influenced by other components. The variable GLS levels of watercress show the need for developing standardized extracts or supplements. Thereby, future clinical trials are provided with a standardized GLS dosage and can overcome logistic and sensory barriers of administering raw watercress.

## Figures and Tables

**Table 1:** Characterization of the study population.

Parameters	
Sex (*n*, females/males)	5/14
Age (years)	26.5 ± 4.3
Weight (kg)	72.9 ± 12.5
Height (m)	1.77 ± 0.09
BMI (kg/m^2^)	23.3 ± 3.5
WHR (females/males)	0.74 ± 0.04/0.84 ± 0.05

BMI = body mass index, WHR = waist-to-hip ratio.

## Appendix A

**Table A1:** Mean concentration of different glucosinolates (GLS) (nmol/g DW) in watercress at different sampling times. The standard deviation represents the values for three technical replicates. Analysis of variance (ANOVA) was performed with Infostat. Means with a common letter are not significantly different.

GLS	Day 1	Day 2	Day 3	Day 4
4-Methoxyglucobrassicin	46.8 ± 2.1 ^a^	70.6 ± 5.0 ^a,b^	101.3 ± 8.3 ^b^	101.5 ± 6.3 ^b^
Glucobrassicin	79.3 ± 2.2 ^a^	87.8 ± 3.2 ^a^	90.5 ± 1.6 ^a^	56.6 ± 1.3 ^a^
Neoglucobrassicin	76.6 ± 1.4 ^a^	76.4 ± 3.7 ^a^	85.5 ± 1.7 ^a^	71.3 ± 3.3 ^a^
Glucoarabishirsutain	213.5 ± 2.6 ^a^	296.4 ± 12.4 ^a,b^	379.6 ± 10.1 ^b^	311.8 ± 11.8 ^a,b^
Gluconasturtiin	4525 ± 45 ^a^	4393 ± 180 ^a^	5966 ± 266 ^a^	5655 ± 196 ^a^
Total	4942 ± 46 ^a^	4924 ± 201 ^a^	6623 ± 285 ^a^	6196 ± 215 ^a^

**Table A2:** Mean contents of antioxidative substances (total flavonoids and phenols, ascorbic acid, α- and γ-tocopherol) and antioxidative capacity (ORAC) in watercress at different sampling times. The standard deviation represents the values for three technical replicates. Analysis of variance (ANOVA) was performed with Infostat. Means with a common letter are not significantly different.

Parameters	Day 1	Day 2	Day 3	Day 4
Total flavonoids (ng CE/g FW)	415 ± 20 ^a^	470 ± 43 ^a,b^	509 ± 49 ^a,b^	529 ± 46 ^b^
Total phenols (ng GAE/g FW)	832 ± 41 ^a^	980 ± 90 ^a,b^	1145 ± 42 ^b^	1178 ± 128 ^b^
Ascorbic acid (µg/g FW)	578 ± 31 ^a^	638 ± 55 ^a^	750 ± 85 ^a^	687 ± 31 ^a^
Carotenoids (µg/g FW)	84.9 ± 8.8 ^a^	108.9 ± 7.7 ^a^	90.3 ± 22.4 ^a^	118.9 ± 16.3 ^a^
α-Tocopherol (µg/g FW)	3.63 ± 0.02 ^a^	5.11 ± 0.11 ^b^	5.58 ± 0.17 ^b^	5.27 ± 0.03 ^b^
γ-Tocopherol (ng/g FW)	70.8 ± 5.5 ^a^	86.9 ± 0.4 ^a^	72.4 ± 3.6 ^a^	60.6 ± 5.1 ^a^
ORAC (µmol TE/g FW)	19.6 ± 2.7 ^a^	20.3 ± 1.1 ^a^	21.4 ± 1.5 ^a^	20.7 ± 2.4 ^a^

CE = catechin equivalents (standard curve was performed with catechin), GAE = gallic acid equivalents (standard curve was performed with gallic acid), ORAC = oxygen radical absorbance capacity, TE = trolox equivalents (standard curve was performed with trolox).

**Table A3:** Effect of acute watercress consumption on blood markers of inflammation (IL-1β, IL-6, IL-10, TNF-α, MCP-1, MMP-9) in ex vivo LPS-stimulated whole blood cultures after high-intensity workout in untrained subjects.

Parameter	Control			Watercress		
	t0	t1	t2	t0	t1	t2
IL-1β (ng/mL)	2.40 ± 0.98 *	2.66 ± 1.26 *	1.98 ± 0.90 ^#^	2.77 ± 0.82 *	3.47 ± 1.45 *^,#^	2.06 ± 0.75 ^#^
IL-6 (ng/mL)	4.09 ± 1.54 *	5.73 ± 1.73 *^,#^	4.29 ± 1.55 ^#^	5.43 ± 1.73 *	7.56 ± 2.25 *^,#^	3.91 ± 1.59 ^#^
IL-10 (pg/mL)	98.0 ± 42.6	144.5 ± 70.3 *^,#^	113.7 ± 73.2 *	107.6 ± 48.1	217.8 ± 103.3 *^,#^	166.6 ± 89.2 *^,#^
TNF-α (pg/mL)	328.3 ± 228.1 *	328.0 ± 140.5 *	296.6 ± 141.1 *	428.0 ± 179.3 *	403.4 ± 163.2 *	205.3 ± 126.1 *^#^
MCP-1 (ng/mL)	3.20 ± 1.73	4.89 ± 2.39 ^#^	3.94 ± 1.57 *^,#^	3.02 ± 1.52	4.67 ± 2.91	3.60 ± 1.96 *^,#^
MMP-9 (ng mL)	86.6 ± 21.5 *	132.2 ± 46.5 ^#^	210.2 ± 50.3 ^#^	105.5 ± 28.3 *	133.9 ± 29.2 ^#^	202.0 ± 45.2 ^#^

t0, pre-exercise; t1, 5 min post-exercise; t2, 3 h post-exercise. * Significant difference between control and watercress. ^#^ Significant difference to previous sampling point.
